# Identification of Wnt/β-catenin- and immune-related genes in hepatic ischemia-reperfusion injury using bulk transcriptomics, single-cell RNA sequencing, and a murine model

**DOI:** 10.3389/fimmu.2026.1745640

**Published:** 2026-09-01

**Authors:** Zhengjun Zhou, Xuanyu Gu, Zhihong Zheng, Yaoli Shao, Kui Tu, Jichang Jiang, Yu Cao, Jin Gu, Lijin Zhao, Hua Yao

**Affiliations:** 1 Department of General Surgery, Digestive Disease Hospital, Affiliated Hospital of Zunyi Medical University, Zunyi, China; 2 Guizhou Provincial Key Laboratory of Digestive System Diseases, Guiyang, China; 3 Department of Critical Care Medicine, Affiliated Hospital of Zunyi Medical University, Zunyi, China

**Keywords:** biomarkers, hepatic ischemia-reperfusion injury, immune response, liver transplantation, Wnt/β-catenin signaling

## Abstract

**Introduction:**

Hepatic ischemia-reperfusion injury (HIRI) is a major complication following liver transplantation (LT), contributing to graft dysfunction and rejection. The roles of the Wnt/β-catenin signaling pathway and immune responses in LT-related HIRI remain incompletely elucidated. This study aimed to screen key genes associated with Wnt/β-catenin signaling scores and immune regulation in HIRI after LT.

**Methods:**

Three LT-related transcriptomic datasets (GSE151648, GSE12720, and GSE189539) were analyzed. Wnt/β-catenin pathway-related genes (WRGs) and immune-related genes (IRGs) were integrated. Weighted gene co-expression network analysis (WGCNA), differential expression analysis, and machine learning algorithms (Boruta and LASSO) were employed to identify hub genes. CIBERSORT was used to perform deconvolution analysis of bulk transcriptomic data and estimate the relative proportions of 22 immune cell types. Regulatory networks of key gene expression, including TF-mRNA and lncRNA-miRNA-mRNA networks, were constructed. Preliminary expression-level validation was performed in a non-transplant murine HIRI model using qPCR, Western blot, histological analysis, and serological assessment.

**Results:**

Four pivotal genes—*HSP90AA1*, *HSP90AB1*, *NFATC1*, and *THBS1*—were identified and validated. These genes were significantly upregulated in post-LT samples and showed good discriminatory potential in the analyzed datasets (AUC > 0.9 in training set, >0.7 in validation set). Functional enrichment linked these genes to cytokine activity and pathways such as the Jak-STAT and chemokine signaling pathways. Immune infiltration analysis revealed correlations with M0/M2 macrophages and resting mast cells. Single-cell sequencing delineated their cell-type-specific expression and differentiation-stage dynamics. In the non-transplant murine HIRI model, the mRNA and protein expression levels of all four genes were significantly upregulated at 6 hours after reperfusion, coinciding with peak injury and supporting their association with acute ischemia-reperfusion injury.

**Conclusion:**

This study identifies *HSP90AA1*, *HSP90AB1*, *NFATC1*, and *THBS1* as crucial biomarkers. Their expression was associated with Wnt/β-catenin signaling scores and immune responses. Results from the non-transplant murine HIRI model supported the upregulation of the four genes during the acute phase of ischemia-reperfusion injury, whereas their potential time-dependent significance and therapeutic value require further validation in LT models and gene functional intervention experiments.

## 1 Introduction

Liver transplantation (LT) is the standard treatment for patients with end-stage liver disease and hepatogenic tumors (1). Although advances in xenotransplantation research are underway, several significant challenges persist, with hepatic ischemia-reperfusion injury (HIRI) being a major concern (2). The ischemic periods and mechanisms of HIRI in liver transplantation donors differ markedly from those in routine hepatectomy procedures. In LT donors, ischemia duration is substantially longer and involves cold and warm ischemia. After transplantation, the donor liver undergoes blood reperfusion by the recipient, accompanied by some degree of immune rejection. In contrast, hepatectomy involves a shorter ischemic period, lacks cold ischemia, and does not involve immune rejection because the recipient’s own blood is restored to the liver.

The critical shortage of hepatic allografts has necessitated the use of extended criteria donors, including older adults (> 60 years), those with moderate to severe hepatic steatosis (> 30%), donation after circulatory death, and grafts with prolonged cold ischemia times (> 12h) requiring normothermic machine perfusion preservation. However, these marginal donors are especially susceptible to HIRI, which is caused by damage sustained during organ procurement, preservation, and surgery (3). HIRI originates through two ischemic pathways: cold and warm ischemia. Cold ischemia occurs during the preservation of the donor liver at low temperatures (0 °C –4 °C) after procurement, which maintains viability during transport until the recipient’s diseased liver is removed. Warm ischemia occurs during implantation, when the cold-preserved donor liver is reintroduced into the recipient’s body and blood reperfusion resumes after reconstruction of the vena cava and portal vein (4). As a sterile local inflammatory response driven by innate immunity, HIRI is a primary contributor to early organ dysfunction and failure after LT. It can cause substantial cellular damage, increasing the risk of graft failure and acute or chronic immune rejection. This exacerbates the critical shortage of life-saving donor organs for liver transplantation. Addressing HIRI is therefore essential for improving transplantation outcomes and expanding the donor pool.

The Wnt signaling pathway in mammals has an evolutionarily conserved tripartite architecture (1): the canonical pathway, responsible for β-catenin-mediated transcriptional activation (2); the noncanonical Wnt/PCP pathway, which coordinates cytoskeletal dynamics through small GTPase-JNK signaling cascades; and (3) the Wnt/Ca²^+^ pathway, which regulates calcium-dependent signaling networks that affect cellular adhesion and transcriptional regulation (5, 6). Among these, the Wnt/β-catenin pathway plays a key role in regulating stem cell pluripotency and determining cell differentiation during development. In hepatocellular carcinoma (HCC), dysregulated Wnt/β-catenin signaling is a critical determinant of adverse clinical outcomes. This abnormal activity reduces the efficacy of HCC therapies by suppressing immune T-cell activation or rendering HCC resistant to immunosuppressive agents (7). However, research on the role of this pathway in ischemia-reperfusion injury of transplanted livers remains limited. Notably, extensive studies have established that Wnt/β-catenin is one of the primary signaling pathways involved in regulating liver homeostasis, regeneration, and tumorigenesis. Based on this evidence, we propose that activation of the Wnt/β-catenin signaling pathway may play a significant role in facilitating repair of ischemia-reperfusion injury in transplanted livers.

Emerging evidence shows that inflammation-inducing immune effector cell activation is a central pathogenic mechanism driving HIRI initiation and progression. However, no effective treatment is currently available to mitigate HIRI after LT (3). Consequently, identifying immune-related therapeutic targets for HIRI after LT is important for alleviating this condition. Notably, Wnt/β-catenin signaling has emerged as a central pathogenic determinant in hepatic ischemia-reperfusion pathogenesis during transplantation procedures (8).

In this study, we integrated conventional transcriptomic and single-cell sequencing data related to LT to screen and identify biomarkers associated with the Wnt/β-catenin signaling pathway and immune responses in LT. We further analyzed the functions and potential molecular mechanisms of these biomarkers in the context of LT. This research may provide theoretical insights and clinical guidance for the prevention and treatment of HIRI after liver transplantation.

## 2 Materials and methods

### 2.1 Data source

Liver transplantation-related datasets were screened using strict inclusion and exclusion criteria. The inclusion criteria were as follows (1): the study was directly related to HIRI (2); the experimental design included control and experimental groups (3); genome-wide transcriptomic expression data were available; and (4) samples were derived from liver tissue. The exclusion criteria were non-liver tissue or non-HIRI model data, too few samples in each group (n < 3), severe data missingness or data that could not be standardized, and datasets without a clear control group or with unclear grouping information. Based on these criteria, a systematic search was performed in the Gene Expression Omnibus (GEO; https://www.ncbi.nlm.nih.gov/geo/) database, and three eligible datasets were included: GSE151648 (platform: GPL21290), GSE12720 (platform: GPL570), and GSE189539 (platform: GPL24676). Other HIRI-related datasets were excluded mainly because of insufficient sample size, lack of liver tissue transcriptomic data, or absence of a clearly defined control group. Among the included datasets, GSE151648 was used as the training set and contained 40 post-LT patient liver tissue samples and 40 pre-LT normal control tissues. GSE12720 was used as the validation set and, after screening, included 11 post-LT reperfused liver tissue samples and 9 pre-LT normal control samples. GSE189539 was used as the single-cell dataset and contained liver tissue samples from 4 post-LT patients at 2 hours after reperfusion and 4 pre-LT normal control liver tissue samples. Basic clinical information for the three datasets is summarized in **Supplementary Table 1**. These datasets served different analytical purposes: GSE151648 was used mainly for candidate gene discovery, GSE12720 for independent validation of expression trends, and GSE189539 for cell-type localization and single-cell expression-feature analysis.

Through systematic curation of Wnt/β-catenin signaling components, 36 pathway regulators (WRGs) were characterized. Key elements included ligand families (*WNT1/3/3A/5A/7A/8*), transmembrane receptors (*FZD1–9* and *LRP5/6*), core regulators (*CTNNB1, GSK3B, AXIN2, APC*), downstream effectors including cell cycle mediators (*CCNA, CDK2*), signaling transducers (*STAT3, EGFR*), extracellular matrix modifiers (*MMP1-3/6-9*), and modulatory BMP family members. Additionally, all immune-related genes (IRGs) were retrieved from the ImmPort database. After duplicate removal, 1,793 IRGs were included, with the complete list provided in **Supplementary Table 2**.

### 2.2 Acquisition of module genes

After clarifying the sources of the study datasets and gene sets, we first evaluated changes in Wnt/β-catenin-related scores in post-LT samples and then identified co-expression modules associated with these scores. WRG scores were calculated for all samples and compared between the post-LT and pre-LT groups in the training set using the ssGSEA algorithm within the GSVA framework (9). Co-expression networks were systematically constructed using WGCNA (v1.72-1) to identify modules showing the strongest associations with WRG scores through topological overlap metric analysis (10, 11). Initially, sample clustering was performed to exclude outlier samples and ensure the reliability of subsequent analyses.

A soft threshold power was then determined to maximize the alignment of intergene interactions with a scale-free distribution. Based on the optimal power value, gene modules were delineated using the hybrid dynamic tree-cutting algorithm, and a module clustering dendrogram was generated. Module-WRG correlations were quantified using Pearson analysis, and two network clusters showing the strongest positive or negative associations (*p* < 0.05, |r| > 0.3) were identified as hub modules. Functional genes within these topological communities (kME > 0.8) were prioritized for downstream characterization.

### 2.3 Screening and functional analysis of candidate genes

After module genes associated with WRG scores, we integrated differentially expressed genes and immune-related genes to progressively narrow the candidate gene set. The DESeq2 package (v1.36.0) (12) was used to identify differentially expressed genes (DEGs) between post-LT and pre-LT samples in the training set, with |log_2_fold change (FC)| > 1 and FDR < 0.05 (13, 14) as thresholds. Volcano plots and heatmaps were generated using the ggplot2 (v3.3.5) and heatmap (v1.0.12) packages, respectively, to display DEG expression. Candidate genes were obtained by crossing DEGs, module genes, and IRGs. To analyze candidate genes and their biological functions, the clusterProfiler package (v4.0.2) (15) was used for Gene Ontology (GO) and Kyoto Encyclopedia of Genes and Genomes (KEGG) pathway enrichment analysis, with *p* < 0.05 and count ≥ 1. Candidate gene-encoded protein interactomes were delineated using the STRING database and Cytoscape-based visualization to construct a high-confidence protein-protein interaction (PPI) network (interaction score threshold > 0.4). Node scores were then calculated using the Degree algorithm via the cytoHubba plugin to assess the importance of candidate genes in the PPI network.

### 2.4 Identification of key genes

To avoid bias introduced by a single screening method, two machine learning algorithms, Boruta and least absolute shrinkage and selection operator (LASSO), were applied to refine the candidate genes. These analyses were performed using the Boruta package (v7.0.0) with default parameters and the glmnet package (v4.1-4) (16, 17), respectively, to obtain feature genes. The intersection of feature genes from Boruta and LASSO was used to identify candidate key genes. The expression of candidate key genes was visualized in the training and validation sets using the ggplot2 package (v3.3.5). Candidate key genes showing significant expression differences between the post-LT and pre-LT groups and consistent expression trends in both datasets were designated key genes. The ability of the key genes to discriminate between post-LT patient samples and pre-LT control samples was assessed by plotting receiver operating characteristic (ROC) curves separately for the two datasets using the pROC package (v1.18.0) (18). Larger areas under the ROC curve (AUC) indicated more accurate prediction (AUC > 70%).

### 2.5 Functional analysis for key genes

After identifying the candidate key genes, we further evaluated their discriminatory ability and potential functional background through reverse model validation, interaction network analysis, and gene set enrichment analysis (GSEA). To evaluate the accuracy of the screened key genes, the key genes were used as inputs to construct a back-propagation (BP) neural network in the training set for reverse verification. First, based on the MSE algorithm, MSE variation curves and error distribution bar graphs for the BP neural network model were generated using the neuralnet package (v1.44.2). Linear regression plots of the model were then drawn to evaluate the error between predicted and actual results. In addition, key genes were entered into the GeneMANIA database to obtain 20 interacting genes and the five most significantly related pathways for the four key genes. Single-gene GSEA for each key gene was performed using the clusterProfiler (v4.0.2) and org.Hs.eg.db (v3.13.0) packages. GSEA used curated databases from MSigDB (GO: c5.go.v7.4.entrez.gmt; KEGG: c2.cp.kegg.v7.4.entrez.gmt). Samples were stratified into high- and low-expression groups according to median key-gene expression thresholds, followed by pathway enrichment analysis using the following significance criteria: |NES| > 1, nominal *p* < 0.05, and FDR *q* < 0.25.

### 2.6 Estimation of relative immune cell proportions based on CIBERSORT

Given the inflammatory and immune characteristics of HIRI, we subsequently analyzed immune cell-related changes and their correlations with key genes. CIBERSORT (v1.06) was used to deconvolve bulk transcriptomes and profile the proportions of 22 immune cell-types in training samples (FDR<0.05). Correlation between the percentages of each immune cell type were then plotted. Based on samples with *p* < 0.05, the ggplot2 package (v3.3.5) was used to compare differences in relative immune cell composition between the post-LT and pre-LT groups. Correlations between key genes and immune cells were analyzed using Spearman correlation, and the results were presented in a heatmap.

### 2.7 Molecular regulatory networks for key genes

To analyze the roles of key genes in expression regulation, regulatory networks of key genes were constructed. Regulatory network inference was conducted using TRRUST v2.0 to map transcription factor (TF)-gene interactions, followed by Cytoscape v3.9.1 visualization of TF-target regulatory architectures. MicroRNAs (miRNAs) binding to key genes were predicted using the miRWalk and miRDB databases, and miRNAs predicted by both databases were selected as key miRNAs. The miRTarBase database was then used to identify long noncoding RNAs (lncRNAs) regulated by miRNAs. Finally, the upstream lncRNA-miRNA-mRNA regulatory network was constructed using Cytoscape.

### 2.8 Single-cell data analysis

To complement the cell-resolution information not provided by bulk transcriptomic data, single-cell data were used to analyze the cell-type distribution and pseudotime characteristics of the key genes. Sequencing data from the single-cell dataset were analyzed using the Seurat package (v4.1.0) (19). The single-cell dataset first underwent quality control (QC) to exclude genes detected in five or fewer low-quality cells, cells with mitochondrial-expressed genes ≥ 20%, cells with erythrocyte-expressed genes ≥ 3%, and cells with less than 200 counts of gene expression, and cells with more than 4,000 total gene counts high-quality cells were then used to perform analysis of variance (ANOVA) to identify 3,000 highly variable genes with significant cell-to-cell expression differences. Next, the single-cell dataset was subjected to dimensionality reduction using principal component analysis (PCA) after gene expression in high-quality cells was normalized using a linear regression model. The overall distribution of cells across samples was assessed for consistency and outlier samples. Dimensionality optimization was performed using Jackstraw permutation testing (1,000 iterations) to identify statistically significant principal components (PCs) (*p* < 0.05). Graph-based clustering was implemented using shared nearest-neighbor modularity (resolution = 0.6, k = 20), with cluster stability validated using silhouette coefficient analysis. Cellular topology was visualized using t-SNE embedding (perplexity = 30, theta = 0.5) with the Rtsne package v0.16. Cell clusters were then annotated based on marker genes reported in the original literature for the single-cell dataset (20). In addition, cell communication in single-cell data was analyzed using the CellPhone DB database. Specifically, the numbers of interacting ligand-receptor pairs and multimers between different cell types were counted. The results of cell communication were then displayed after filtering with a threshold of *p* ≤ 0.05.

To further reveal the relationship between key genes and LT, the expression of each key gene in each cell type was analyzed. Finally, pseudo-temporal analysis was performed using the Monocle 2 algorithm in the monocle package (v2.24.1) to project different cell types onto a root and construct a single-cell trajectory map. Correlations between key genes and WRG scores were also calculated to assess their relationship.

### 2.9 Validation of key genes in a murine model of hepatic ischemia-reperfusion injury

To further validate key gene expression in HIRI, a murine HIRI model was established. Adult male mice were anesthetized by intraperitoneal injection of ready-to-use 1.25% tribromoethanol solution (Nanjing Aibei Biotechnology Co., Ltd., Cat# M2910) at a dose of 0.2 mL/10 g body weight. Laparotomy was then performed, and ischemia was induced by occluding the left and median liver lobes (approximately 70% of the hepatic blood supply) for 1 hour. The abdomen was closed to initiate reperfusion, and tissue samples were collected at 0, 6, and 12 hours after reperfusion. At each time point, mice were euthanized by isoflurane inhalation (RWD Life Science Co., Ltd., Cat# R510-22-10) at a flow rate of 0.41 mL/min, followed by cervical dislocation after anesthesia induction. Blood samples were collected from mice via the retro-orbital venous plexus, allowed to stand at room temperature for 30 min, and centrifuged at 3,000 rpm for 15 min at 4 °C to separate serum. Serum alanine aminotransferase (ALT) and aspartate aminotransferase (AST) levels were measured using a fully automatic biochemical analyzer (Servicebio, Cat# GH-100) to evaluate liver function before and after HIRI. Concurrently, liver tissues were processed for H&E staining and TUNEL assay to confirm successful establishment of the model.

After model validation, total RNA was extracted from liver tissues in each group using TRIzol reagent (Beyozol, Cat# R0011). RNA concentration and purity were determined using a Thermo Scientific NanoDrop One spectrophotometer. Subsequently, 1 μg of total RNA was reverse-transcribed into cDNA using a reverse transcription kit (Beyotime, Cat# D7180S) according to the manufacturer’s instructions. Quantitative real-time PCR (qPCR) was performed on a CFX-96 qPCR system using SYBR Green master mix (Bio-Rad, Cat# 1725121) with cDNA as the template to measure the expression of *HSP90AA1*, *HSP90AB1*, *NFATC1*, and *THBS1*. The thermal cycling conditions were 95 °C for 30 s, followed by 40 cycles of 95 °C for 5 s and 60 °C for 30 s. *GAPDH* was used as the internal reference, and relative expression levels were calculated using the 2^−ΔΔCt^method. Primer sequences and raw Ct values are provided in **Supplementary Table 3** and **Supplementary Table 4**, respectively. For Western blot analysis, total protein was extracted from the frozen liver tissues of the HIRI model using RIPA lysis buffer. After BCA quantification, equal amounts of protein were separated by SDS-PAGE and transferred to PVDF membranes. Membranes were blocked and then incubated with primary antibodies against THBS1 (Proteintech, 18304-1-AP), HSP90AA1 (Proteintech, 13171-1-AP), HSP90AB1 (Proteintech, 11405-1-AP), NFATC1 (Proteintech, 66963-1-Ig), and β-actin (Proteintech, 66009-1-Ig) as the loading control. After incubation with HRP-conjugated secondary antibodies, bands were visualized using ECL and quantified with ImageJ. Quantified results are presented in **Supplementary Table 5**, and full-length blots are shown in **Supplementary Figure 1**. This study was approved by the Animal Welfare and Ethics Committee of Zunyi Medical University (Approval No. ZMU21-2503-602).

### 2.10 Statistical analysis

All statistical analyses were performed using R v4.2.2 for omics data and GraphPad Prism (v10.1.2) for murine experimental data. For omics data, a significance threshold of p < 0.05 with Benjamini-Hochberg FDR correction was applied. Murine data are presented as the mean ± SD and were analyzed by one-way ANOVA after confirmation of normality and homogeneity of variance, with Tukey’s or Dunnett’s *post hoc* test used as appropriate. A p value < 0.05 was considered statistically significant.

## 3 Results

### 3.1 WRG score-related modules, candidate gene screening, and identification of four key genes

**Figure 1:**
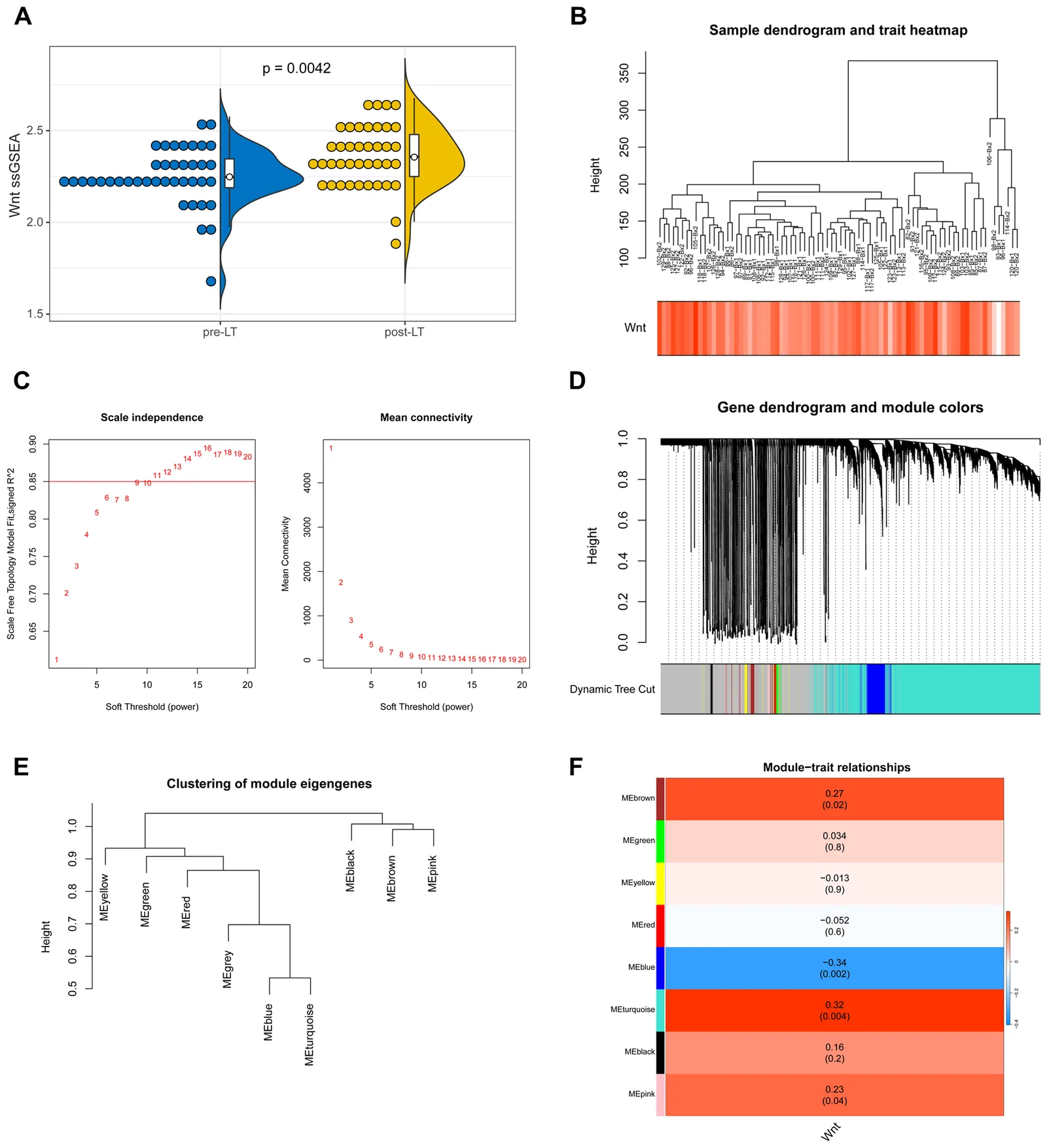
Analysis of liver transplantation-related gene modules in the training set. **(A)** Comparison of WRG scores before and after liver transplantation in the training set. **(B)** Clustering results of samples showing no outliers in the training set. **(C)** Determination and analysis of the optimal soft-thresholding power in the training set. **(D–E)** Analysis of gene module division via dynamic tree cutting in the training set. **(F)** Correlation analysis between WRG scores and gene modules in the training set.

First, in the training set, WRG scores were significantly higher in post-LT samples than in pre-LT samples (**Figure 1A**). Sample clustering showed no outlier samples (**Figure 1B**). The best power value was determined to be 11, at which R^2^ = 0.9, indicating that the network approximated a scale-free distribution (**Figure 1C**). Next, nine gene modules were identified using dynamic tree cutting (**Figures 1D, E**). Based on correlations between WRG scores and the nine modules, the blue module (r = -0.34, *p* = 0.002, 885 genes) and turquoise module (r = 0.32, *p* = 0.004, 6,299 genes) were selected (**Figure 1F**). Together, these modules contained 7,184 module genes.

**Figure 2:**
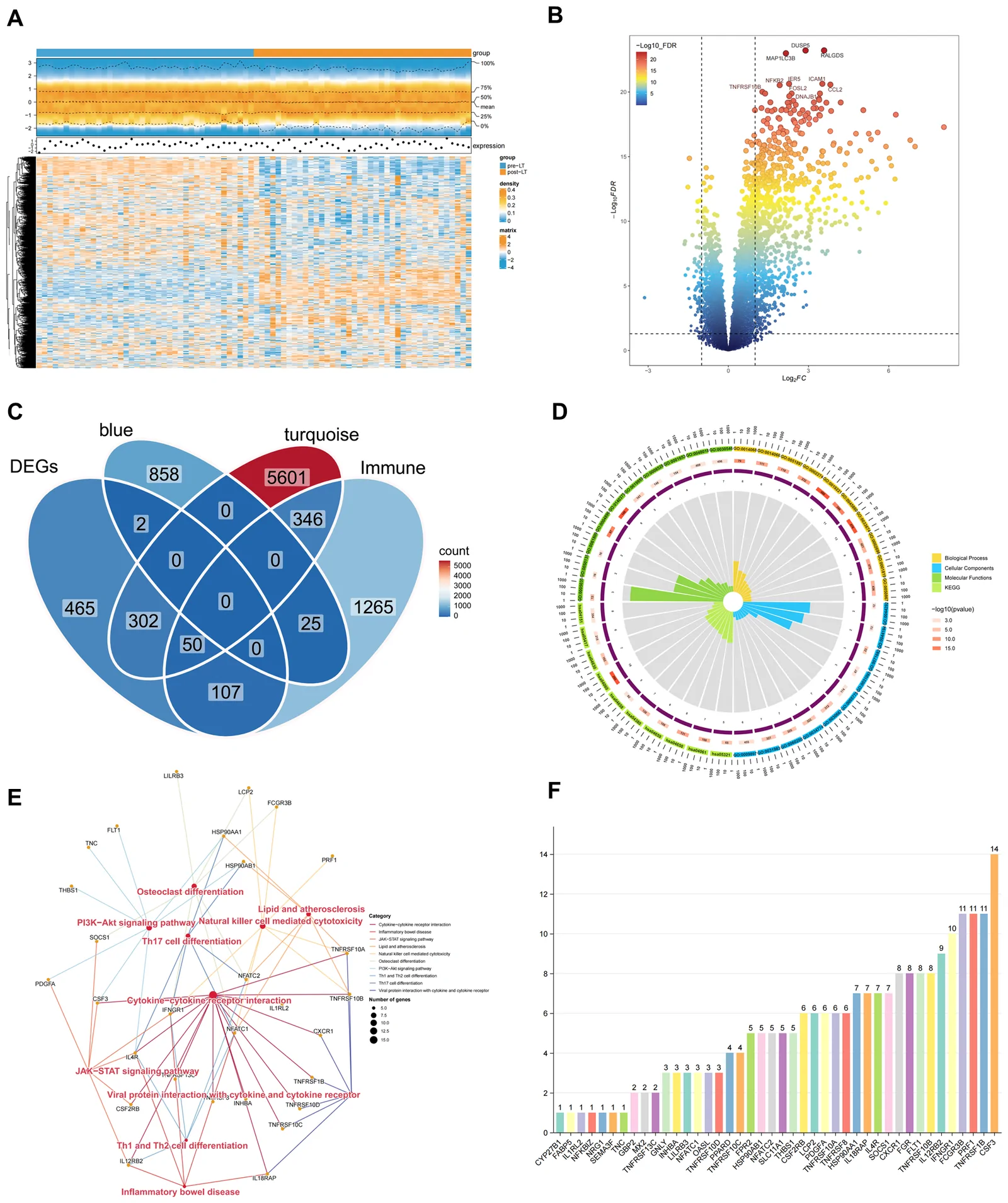
Candidate gene screening and functional analysis in the liver transplantation training set. **(A)** Heatmap of DEG expression (post-LT vs. pre-LT, training set). **(B)** Volcano plot of DEGs (post-LT vs. pre-LT, training set). **(C)** Venn diagram for candidate biomarker identification. **(D)** Circular plot of GO functional annotation. **(E)** KEGG pathway enrichment network. **(F)** PPI network of candidate genes, with CSF3 highlighted.

Based on these results, module genes associated with WRG scores were used for subsequent candidate gene screening to ensure that the following analyses focused on Wnt/β-catenin-related transcriptional features. DEGs were then identified. Between the post-LT and pre-LT groups in the training set, 926 DEGs were identified, including 862 upregulated and 66 downregulated genes (**Figures 2A, B**). Venn diagram intersection of the 926 DEGs and 7,184 module genes identified 50 candidate biomarkers (**Figure 2C**). Functional annotation revealed that these genes were enriched in 647 biological processes, 32 cellular components, and 70 molecular functions (GO terms), as well as 26 KEGG pathways, including “cytokine-cytokine receptor interaction” (**Figures 2D, E**). A PPI network was then constructed, and CSF3 showed high importance in the network (**Figure 2F**).

**Figure 3:**
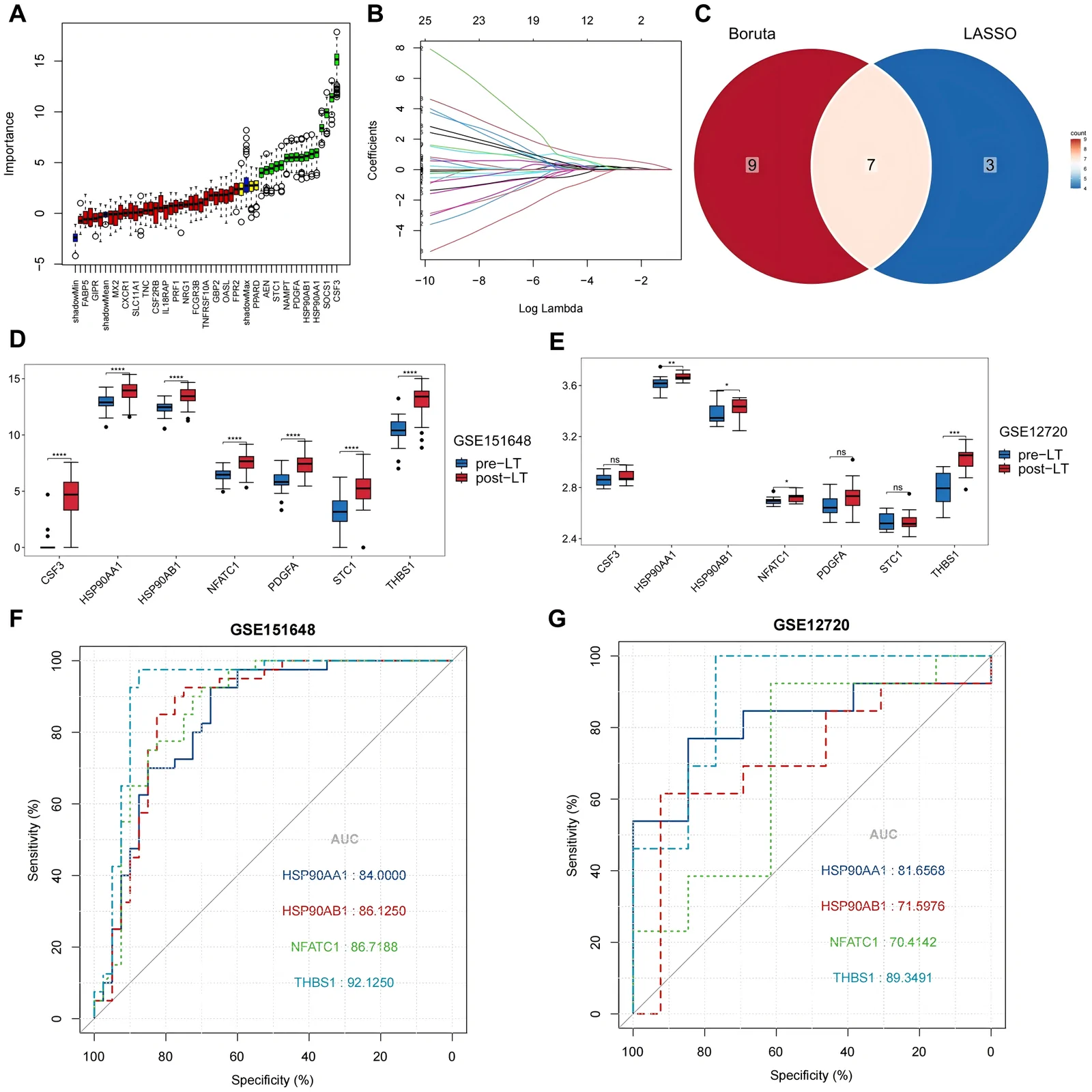
Identification and validation of key genes in liver transplantation datasets. **(A)** Feature gene screening using the Boruta algorithm. **(B)** Feature gene screening using the LASSO algorithm. **(C)** Venn diagram for candidate key gene identification. **(D)** Differential expression of candidate key genes in GSE151648 (training set). **(E)** Differential expression of candidate key genes in GSE12720 (validation set). **(F)** ROC curves of key genes in the training set. **(G)** ROC curves of key genes in the validation set. *p < 0.05, **p < 0.01, ***p < 0.001, ****p < 0.0001, ns = not significant (p>0.05).

After the 50 candidate biomarkers were obtained, feature selection was performed using two machine learning algorithms to identify more stable candidate key genes. Among the 50 candidate genes, 16 and 10 feature genes were screened using the Boruta and LASSO algorithms, respectively (**Figures 3A, B**). Intersecting the feature genes from the two machine learning algorithms yielded seven candidate key genes (*THBS1*, *CSF3*, *HSP90AB1*, *NFATC1*, *PDGFA*, *HSP90AA1*, and *STC1*) for further analysis (**Figure 3C**). All candidate key genes were significantly differentially expressed in the training set, and four candidate key genes were significantly differentially expressed between the post-LT and pre-LT groups in the validation set. *HSP90AA1*, *HSP90AB1*, *NFATC1*, and *THBS1* were upregulated in the post-LT group in both datasets (**Figures 3D, E**). In the training set, these four candidate key genes had AUC values greater than 90%. In the validation set, their AUC values exceeded 70%, indicating that HSP90AA1, HSP90AB1, NFATC1, and THBS1 had good sample-discriminatory potential within the datasets analyzed. This result reflected statistical discriminatory ability within the datasets and should not be considered equivalent to clinical diagnostic performance (**Figures 3F, G**). In summary, four key genes (*HSP90AA1*, *HSP90AB1*, *NFATC1*, and *THBS1*) were associated with LT.

### 3.2 Preliminary validation and functional characterization of the four key genes in HIRI

**Figure 4:**
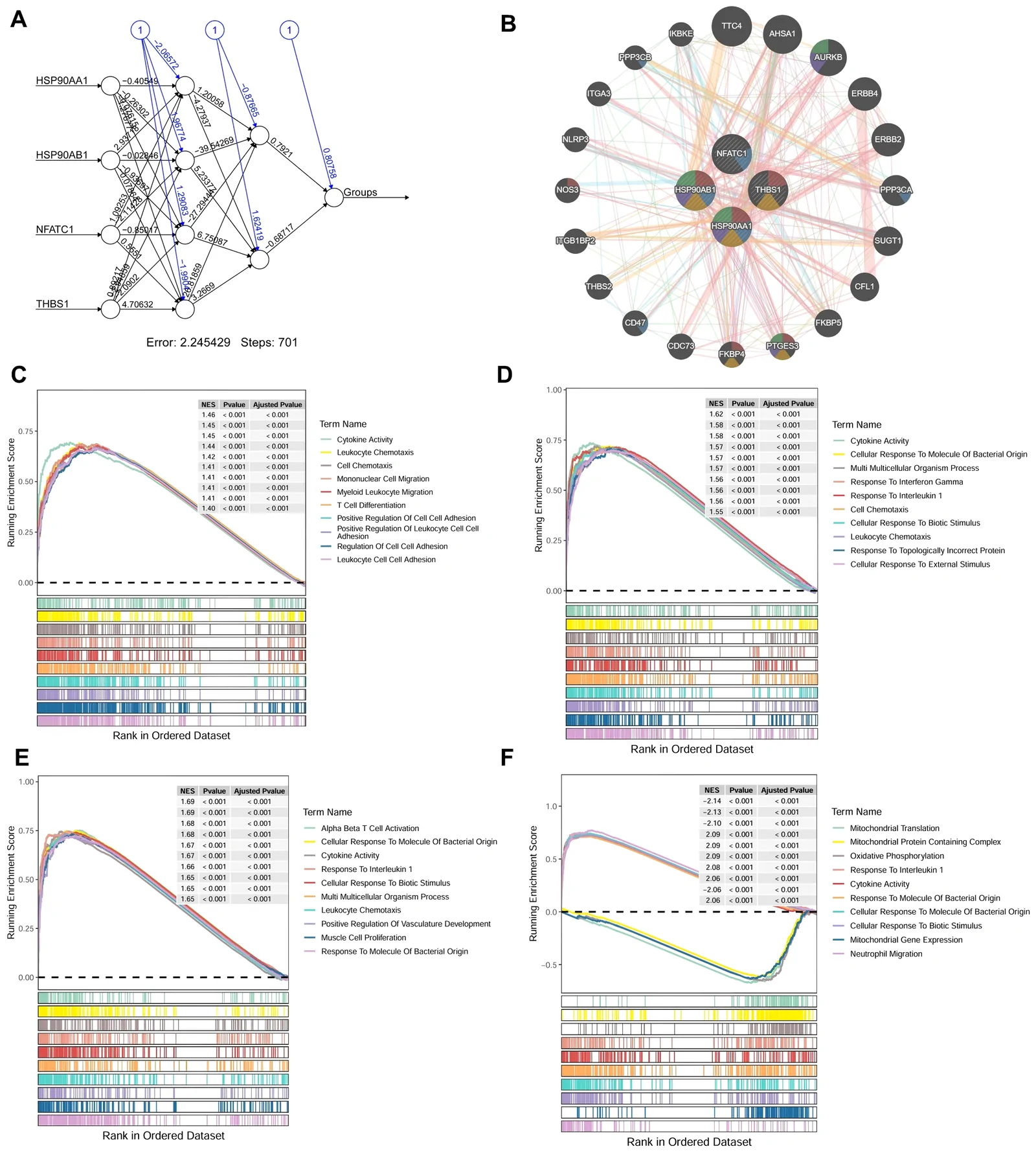
Functional validation and enrichment analysis of key genes. **(A)** BP neural network model validation based on the correlation between predicted and actual values. **(B)** Key gene interaction network and related pathways from the GeneMANIA database. **(C–F)** ssGSEA-based GO and KEGG enrichment results for key genes.

After identifying the four candidate key genes, the analysis shifted from screening to validation and interpretation, including assessment of their discriminatory potential, model performance, and related functional pathways. For the BP neural network model, the correlation between predicted and actual values was 0.9156, with an error of 0.04, suggesting that the four key genes showed good validation performance (**Figure 4A**). Based on the GeneMANIA database, 20 genes interacting with the key genes and the most significantly related pathways are shown in **Figure 4B**. The results indicated that the four key genes were significantly associated mainly with the “Fc receptor signaling pathway,” “regulation of telomerase activity,” and related pathways. Enrichment results for *HSP90AA1*, *HSP90AB1*, *NFATC1*, and *THBS1* were obtained separately using ssGSEA based on GO and KEGG (**Figures 4C–F**). For GO terms, all four key genes were enriched in “cytokine activity.” *HSP90AA1*, *HSP90AB1*, and *NFATC1* were enriched in “leukocyte chemotaxis,” whereas *HSP90AB1*, *NFATC1*, and *THBS1* were enriched in “response to interleukin 1.” For KEGG pathways, *HSP90AA1*, *HSP90AB1*, and *THBS1* were enriched in Jak-STAT, MAPK, chemokine signaling, and related pathways.

### 3.3 Immune-related correlations and regulatory network analysis of key genes in HIRI

**Figure 5:**
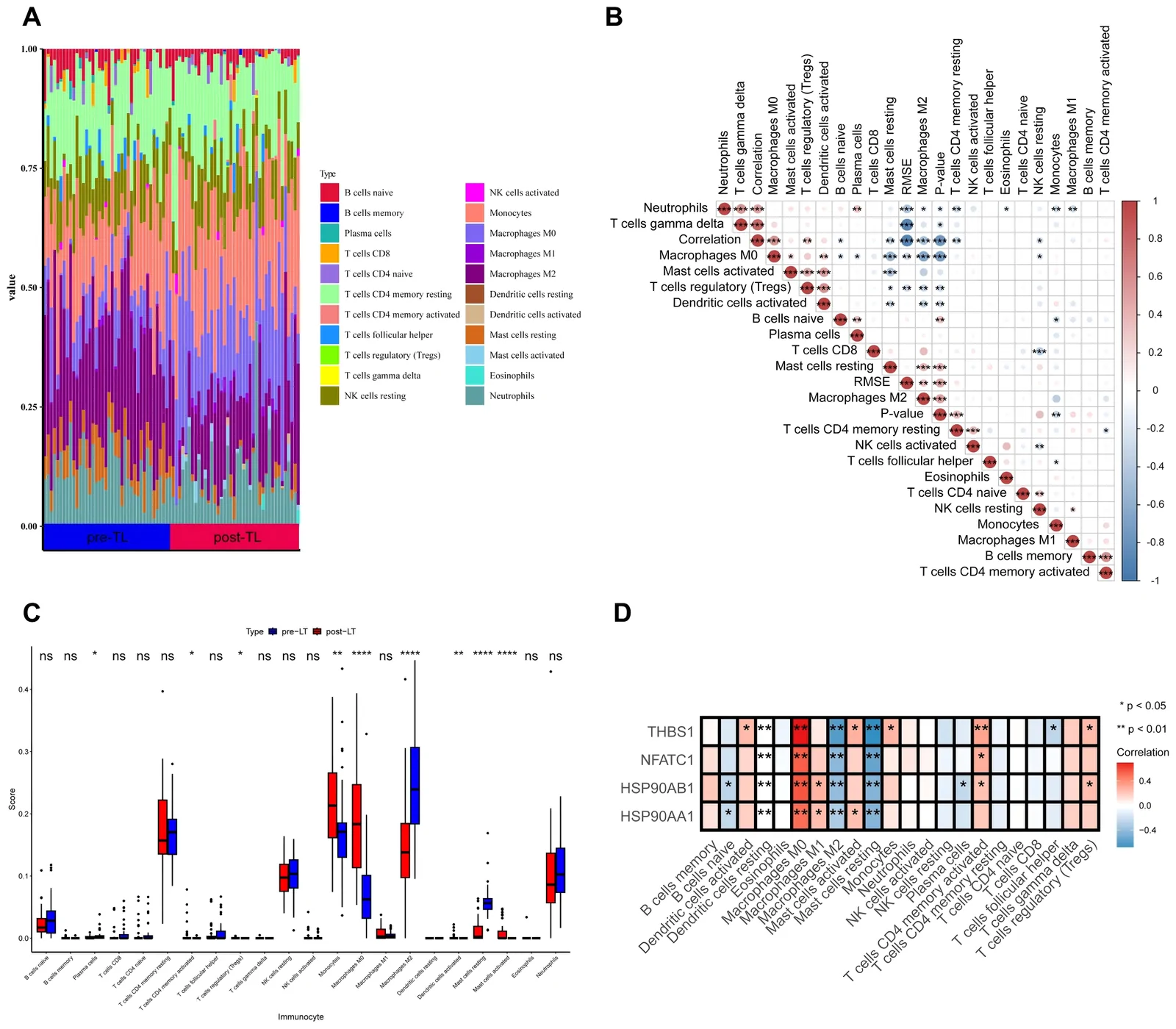
Immune cell infiltration and correlation analysis in liver transplantation. **(A)** Heatmap showing the infiltration percentages of immune cell types (pre-LT vs. post-LT, training set). **(B)** Correlation matrix of immune cell infiltration. **(C)** Differential immune cell infiltration between pre-LT and post-LT groups. **(D)** Correlations between key genes and immune cell subsets. *p < 0.05, **p < 0.01, ***p < 0.001, ****p < 0.0001, ns = not significant (p>0.05).

In the training set, the relative proportions of 22 immune cell types and their correlations were comprehensively displayed, providing insight into the immune cell composition landscape within the dataset (**Figures 5A, B**). Nine immune cell types differed markedly between the pre-LT and post-LT groups (**Figure 5C**). Correlation analysis revealed a notable positive correlation between M0 macrophages and the four key genes, whereas M2 macrophages and resting mast cells showed notable negative correlations with each key gene (**Figure 5D**). These immune cell subsets may therefore be associated with immune-related changes during LT-related HIRI, encompassing aspects such as graft acceptance, immune-mediated complications, and the restoration of normal immunological homeostasis.

**Figure 6:**
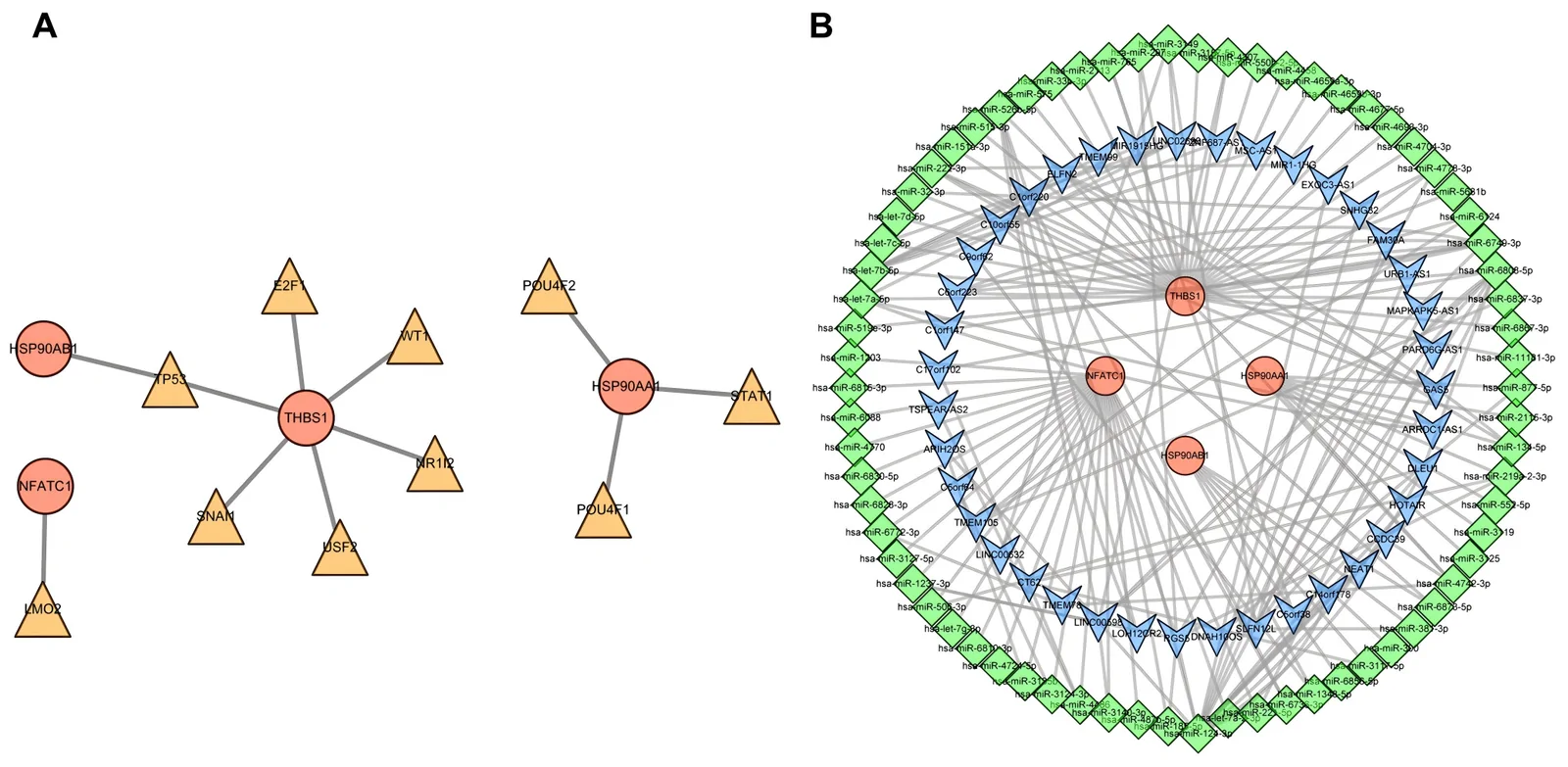
Key gene-mediated regulatory networks of TFs, miRNAs, and lncRNAs. **(A)** TF-mRNA network of key genes. **(B)** lncRNA-miRNA-mRNA network of key genes.

After CIBERSORT analysis suggested that the candidate key genes were associated with immune cell composition, transcriptional and post-transcriptional regulatory networks were constructed to explore potential upstream regulatory clues. Bioinformatics analysis predicted 10 TFs, 70 miRNAs, and 39 lncRNAs associated with the key genes. The TF-mRNA network showed that *TP53* was predicted as a TF for *HSP90AB1* and *THBS1*, and three TFs were predicted for *HSP90AA1* (**Figure 6A**). A total of 70 miRNAs corresponding to the four key genes were predicted from two databases, and 39 lncRNAs were predicted based on these 70 miRNAs. Thus, the lncRNA-miRNA-mRNA network consisted of 70 miRNAs, 39 lncRNAs, and four key genes, including hsa-miR-222-3p-GAS5-THBS1, hsa-miR-124-3p-DLEU1-NFATC1, and hsa-miR-765-C5orf38-THBS1 (**Figure 6B**). This lncRNA-miRNA-mRNA network was constructed based on database predictions and provided candidate molecular resources for subsequent exploration of upstream post-transcriptional regulatory mechanisms of the four key genes in HIRI. This network should currently be regarded as a suggestive screening tool, and its regulatory relationships require validation through experiments such as RNA pull-down assays or dual-luciferase reporter assays.

### 3.4 Single-cell RNA sequencing analysis revealed the cellular landscape of HIRI and cell-type features of key genes

**Figure 7:**
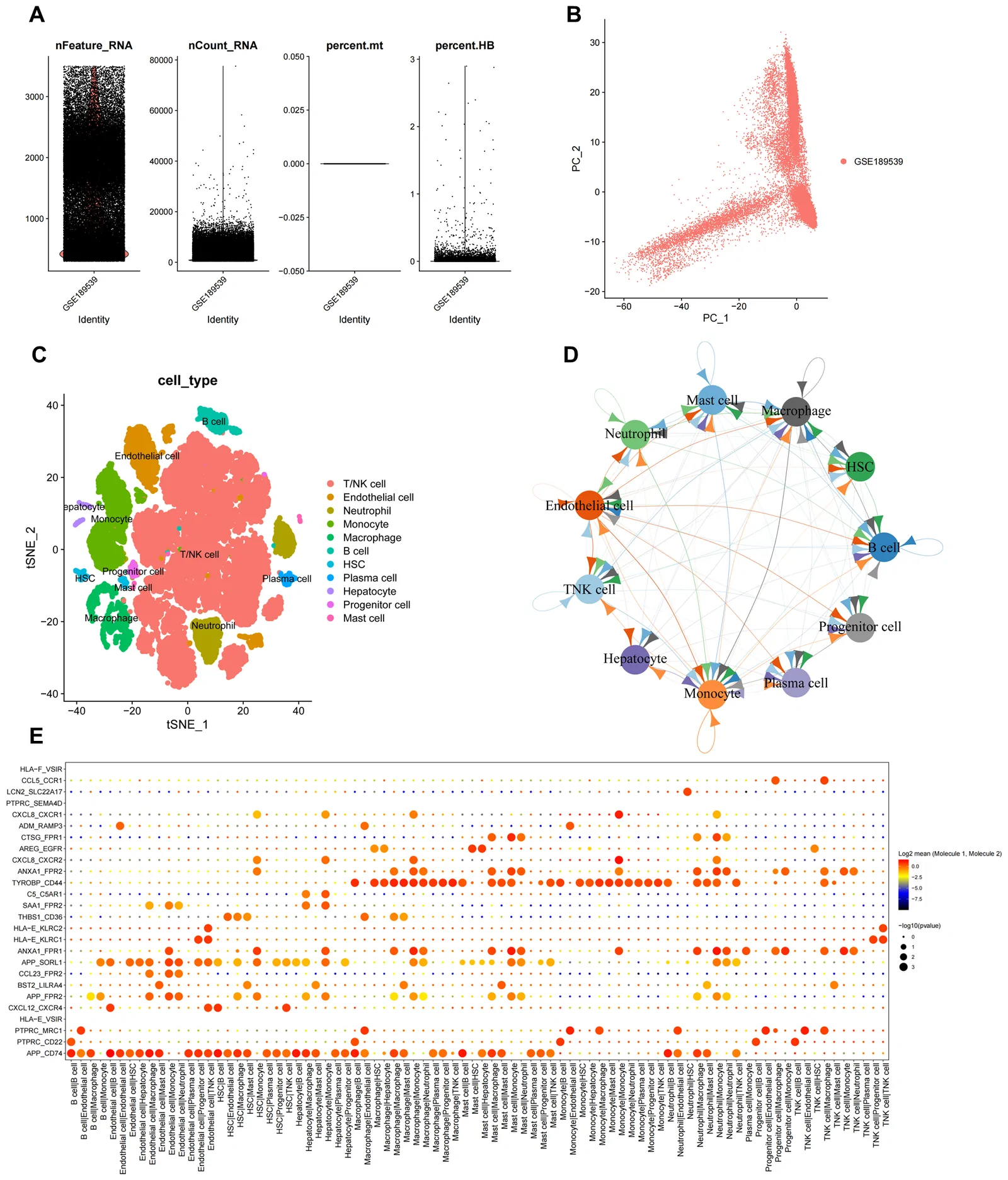
Single-cell dataset bioinformatics analysis and cell-type delineation. **(A)** Quality control results for the single-cell dataset. **(B)** Identification of variable genes for PCA. **(C)** t-SNE visualization of cell clusters. **(D)** Cell-type annotation results. **(E)** Cell communication analysis of ligand-receptor interactions.

To further interpret the bulk transcriptomic screening results at single-cell resolution, we analyzed the GSE189539 single-cell RNA sequencing data. After stringent QC measures on the single-cell dataset, 55,901 high-fidelity cells were retained (**Figure 7A**). Subsequently, 3,000 highly variable genes were identified for PCA (**Figure 7B**). A total of 40 PCs were selected, and 27 distinct cell clusters were identified and visualized using t-SNE (**Figure 7C**). Some clusters were assigned to the same cell type based on shared marker genes. After cellular annotation, 11 cell types were identified: T or natural killer (NK) (T/NK) cells, endothelial cells, neutrophils, monocytes, macrophages, B cells, hematopoietic stem cells (HSCs), plasma cells, hepatocytes, progenitor cells, and mast cells (**Figure 7D**). Marker genes for different cell types and clusters, as well as the expression of marker genes in each cell type and cluster, are shown in **Supplementary Table 6** and **Supplementary Figures 2**, **3**. These results established a single-cell-level cellular composition landscape of LT-related HIRI and provided a basis for further analysis of the cellular features and biological implications of the key genes.

After defining the major cell types, potential communication relationships among different cell populations were further analyzed. Cell communication analysis identified 26 ligand-receptor and multimeric interactions; for example, B cells communicated with macrophages, endothelial cells, and monocytes (**Supplementary Figure 4**). Ligand-receptor and multimeric interactions are shown for all cell afferent and efferent signals, the, with redder dots indicating stronger ligand-receptor, multimeric action (**Figure 7E**).

**Figure 8:**
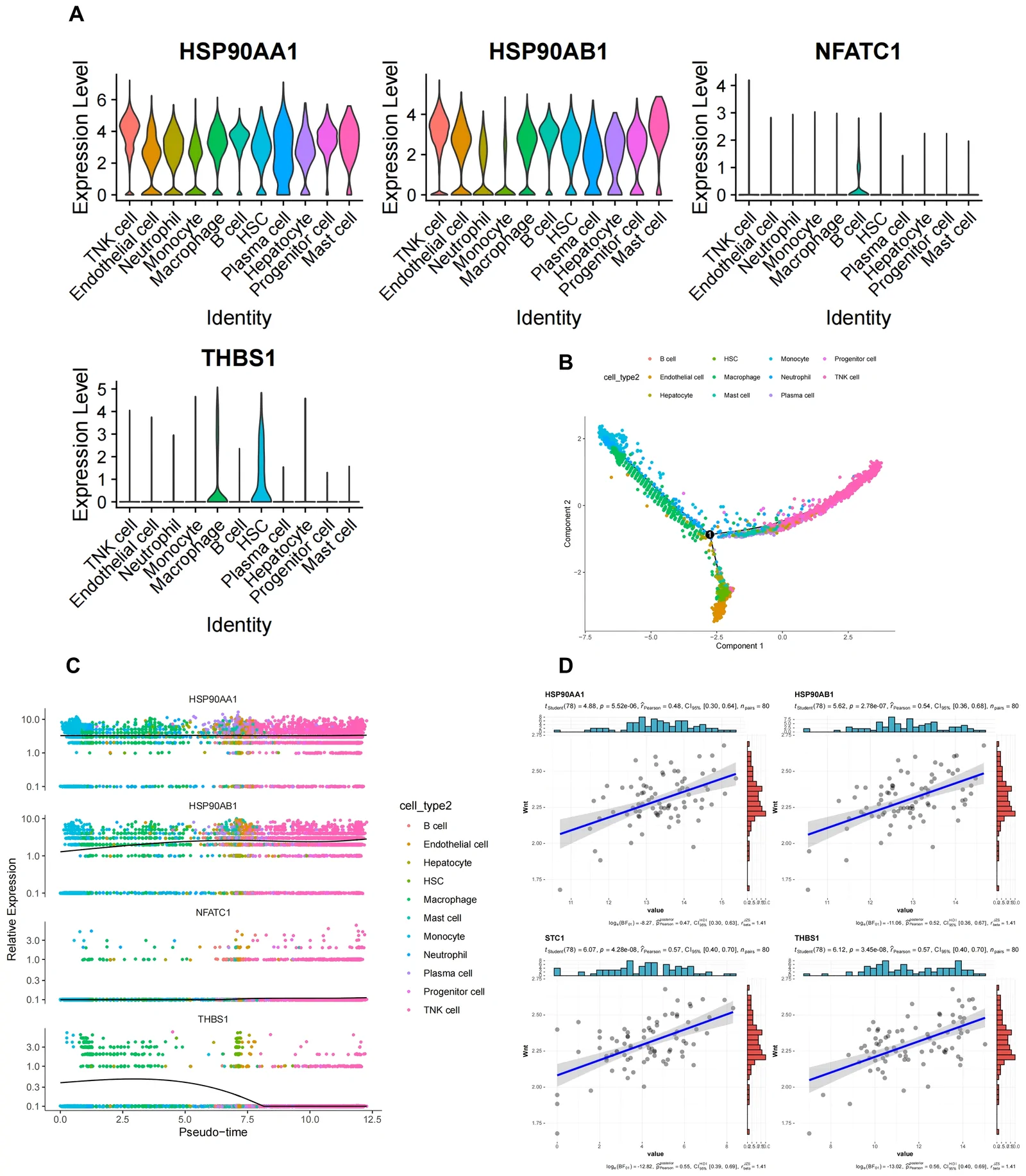
Expression analysis of the four key genes across cell types. **(A)** Expression levels of key genes across cell types. **(B)** Single-cell trajectory map of cell differentiation. **(C)** Expression dynamics of key genes during cell differentiation. **(D)** Correlations between key genes and the Wnt signaling pathway.

On this basis, the expression characteristics of the four candidate key genes were further localized across different cell types. Expressions of key gene in each cell type showed that *HSP90AA1* and *HSP90AB1* were expressed in multiple cell types, *NFATC1* was highly expressed mainly in B cells, and THBS1 was highly expressed in macrophages and HSCs (**Figure 8A**). In the single-cell trajectory map, cell differentiation proceeded from left to right, with no obvious change in *HSP90AA1* or *NFATC1* expression during differentiation, high *THBS1* expression at the predifferentiation stage, and high *HSP90AB1* expression at the late differentiation stage (**Figures 8B, C**). These results suggested that different candidate key genes may correspond to distinct cell types or cellular states, although their specific functions require further experimental validation.

Finally, to further connect the single-cell findings with the Wnt/β-catenin-related analysis, we calculated the correlations between the four candidate key genes and Wnt signaling pathway scores. All four candidate key genes were significantly positively correlated with Wnt signaling pathway scores (**Figure 8D**), suggesting a statistical association between them. This result mainly reflected expression-level correlation and did not demonstrate a direct regulatory relationship between the four genes and the Wnt signaling pathway.

### 3.5 Experimental validation of key genes in a hepatic ischemia-reperfusion injury model

**Figure 9:**
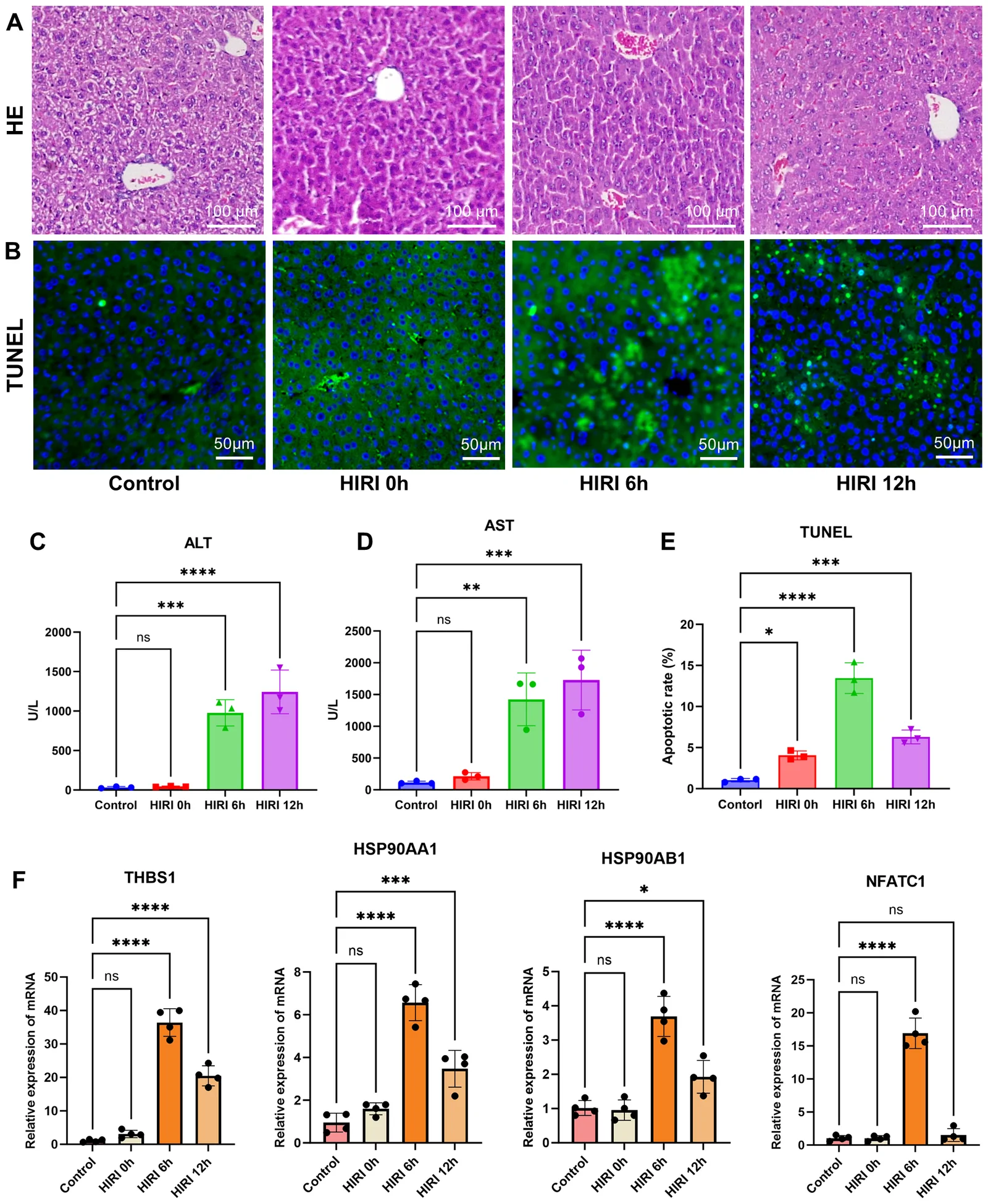
Validation of key gene expression in the HIRI model. **(A)** H&E staining showing hepatocyte necrosis and microvascular injury at 6–12 h after reperfusion. **(B)** TUNEL staining showing apoptotic nuclei spreading from central to peripheral zones. **(C–D)** Increased serum ALT and AST levels after reperfusion. **(E)** Peak apoptosis at 6 h after reperfusion. **(F)** Significant upregulation of key genes (*THBS1*, *HSP90AA1*, *HSP90AB1*, and *NFATC1*) at 6 h. Data are presented as the mean ± SEM. ns, not significant; *p < 0.05; **p < 0.01; ***p < 0.001; ****p < 0.0001 versus control.

Based on the above computational analysis results, we further performed preliminary expression-level validation in a non-transplant murine HIRI model. To validate successful establishment of the HIRI model, H&E staining was first performed on liver tissues. The results (**Figure 9A**) showed marked hepatocyte necrosis in the ischemia-reperfusion (I/R) group at 6–12 hours after reperfusion, accompanied by microcirculatory disturbances, including endothelial cell swelling and detachment, exposure of the basement membrane, and subsequent platelet aggregation, and microthrombus formation. TUNEL staining was then conducted to assess apoptosis (**Figure 9B**). TUNEL-positive nuclei were predominantly located in the ischemic central area, with positive signals spreading to peripheral regions as reperfusion time increased. Positive staining was also observed around portal areas, particularly in regions with severe inflammatory responses. These nuclei exhibited typical apoptotic morphological features, including shrinkage, fragmentation, and a granular or clustered appearance. In severely damaged areas, TUNEL-positive cells were distributed in clusters or diffusely and displayed bright green fluorescence under fluorescence microscopy.

**Figure 10:**
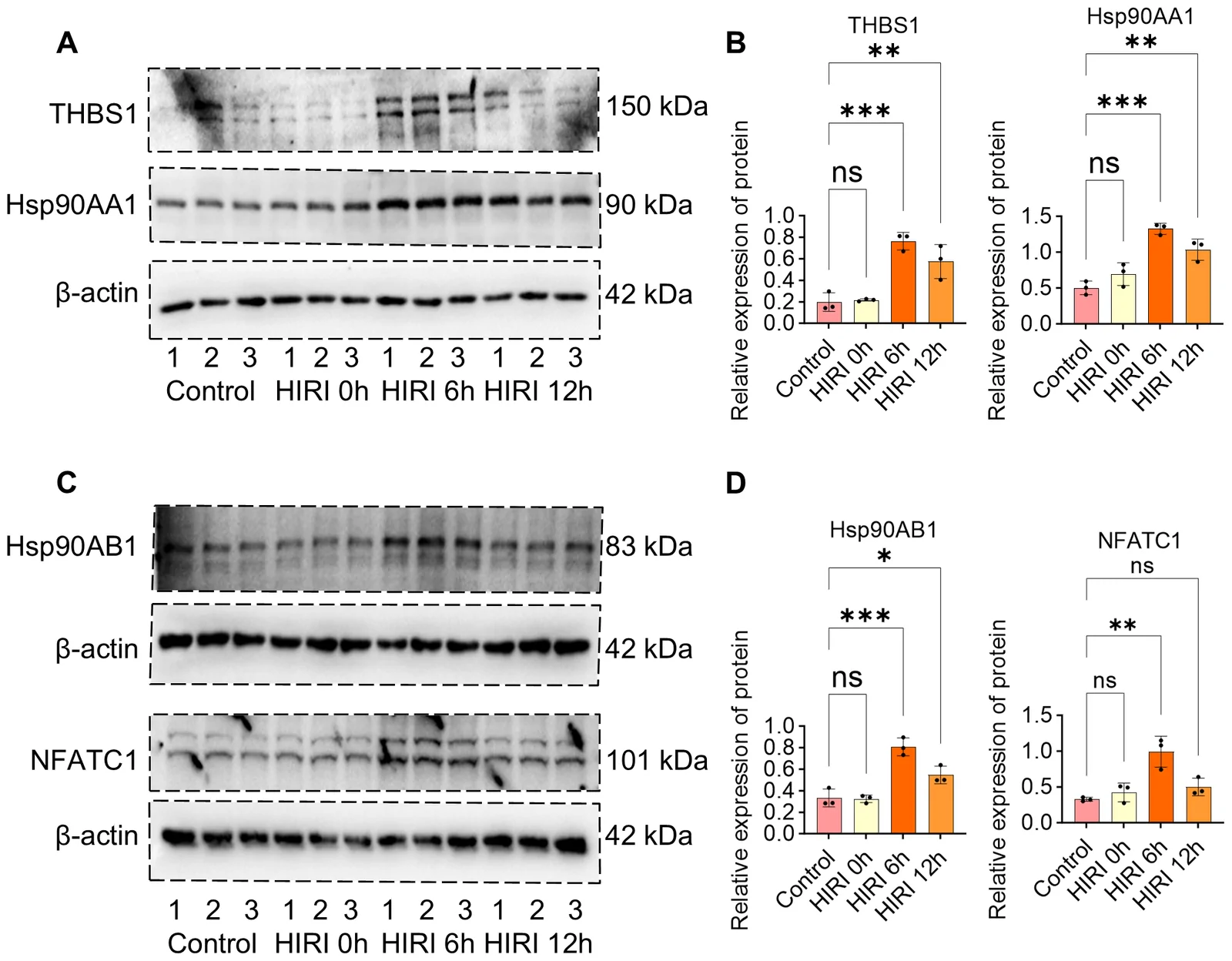
Protein expression validation of key genes in the HIRI model. **(A)** Representative immunoblots for THBS1 and HSP90AA1. **(B)** Densitometric quantification of THBS1 and HSP90AA1 normalized to β-actin. **(C)** Representative immunoblots for HSP90AB1 and NFATC1. **(D)** Densitometric quantification of HSP90AB1 and NFATC1 normalized to β-actin. Protein levels were measured at 0, 6, and 12 h after reperfusion; expression peaked at 6 h and declined by 12 h, consistent with the qPCR results. Data are presented as the mean ± SEM. ns, not significant; *p < 0.05; **p < 0.01; ***p < 0.001 versus control.

Serological analysis (**Figures 9C, D**) indicated that serum levels of alanine aminotransferase and aspartate aminotransferase were consistently elevated after reperfusion, suggesting significant liver function impairment. Moreover, the number of TUNEL-positive cells and the apoptosis rate peaked at 6 hours after reperfusion (**Figure 9E**), confirming this time point as the peak of apoptotic activity. Together, these results demonstrate successful induction of the HIRI model. Based on this established model, we examined the expression of four key genes (*THBS1*, *HSP90AA1*, *HSP90AB1*, and *NFATC1*) in liver tissues (**Figure 9F**). At 0 hours of reperfusion, their expression levels did not differ significantly from those in the control group. However, at 6 hours after reperfusion, all four genes were significantly upregulated and reached peak expression, a trend consistent with the progression of hepatocellular injury. To further corroborate these findings at the protein level, we performed Western blot analysis (**Figure 10**). Consistent with the qPCR results, protein expression of *THBS1*, *HSP90AA1*, *HSP90AB1*, and *NFATC1* showed no significant change at 0 hours of reperfusion, reached peak levels at 6 hours, and began to decline by 12 hours(**Figures 10A–D**). This protein expression pattern mirrored the transcriptional dynamics, further supporting the consistent upregulation of these four genes during the acute phase of HIRI in non-transplant mice and providing preliminary experimental evidence for their expression changes associated with ischemia–reperfusion injury. However, their functional roles in LT-related HIRI require further confirmation using liver transplantation models and gene function intervention.

## 4 Discussion

In this study, we integrated transcriptomic and single-cell data and identified four pivotal biomarkers—*HSP90AA1*, *HSP90AB1*, *NFATC1*, and *THBS1*—associated with the Wnt/β-catenin signaling pathway and immune regulation in LT. Critically, their dynamic expression patterns observed in bioinformatics analyses were preliminarily supported by experimental validation in a murine HIRI model. Specifically, qPCR and Western blot analysis showed that all four genes were significantly upregulated 6 hours after reperfusion, coinciding with peak hepatocellular injury and apoptosis, suggesting that their expression may reflect the acute injury response. Single-cell trajectory analysis further suggested that *THBS1* expression was associated with the early differentiation stage, whereas *HSP90AB1* appeared to be more active at later stages, indicating that these genes may exhibit distinct temporal expression patterns.

*HSP90AB1* is a member of the heat shock protein (HSP) superfamily and primarily functions as a molecular chaperone. It binds to client proteins to facilitate proper folding and maintain protein stability (21–23). Through the coordinated action of multiple pathways, it regulates key biological processes, including gene expression, the cell cycle, and cell proliferation (24). Heat shock protein 90α (Hsp90α), encoded by *HSP90AA1*, belongs to the stress-induced subtype of HSP90 molecular chaperones (25). Prior research has highlighted the importance of maintaining a balance between regeneration and tumorigenesis in biological and physiological processes (26). In this study, *HSP90AA1* and *HSP90AB1* were upregulated in LT-related datasets, and their expression levels were positively correlated with Wnt/β-catenin pathway scores, suggesting a potential association between the HSP90 family and Wnt-related transcriptional status in HIRI. This upregulation trend was supported by public data analyses and *in vivo* animal experiments, further indicating that these molecules may be involved in the pathogenesis of HIRI after LT. Thus, these genes represent potential therapeutic targets that warrant further investigation. These results are consistent with previously established roles of HSP90 in other complex pathophysiological processes, including viral infection and tumor development (27). In addition, research on gastric cancer demonstrated that *HSP90AB1*, a member of the HSP90 family, interacted with LRP5, an essential co-receptor in the Wnt/β-catenin signaling pathway. This molecular interplay suppressed LRP5 ubiquitination, thereby triggering canonical Wnt/β-catenin pathway hyperactivation that orchestrates epithelial-mesenchymal transition initiation and malignant propagation (28). These findings provide indirect evidence for a possible molecular link between HSP90 and Wnt/β-catenin signaling, but whether this association can be extrapolated to LT-related HIRI requires dedicated experimental validation.

The *NFATC1* gene product is a key component of the nuclear factor of activated T cells (NFAT) DNA-binding transcription complex (29, 30). This complex consists of at least two elements: a cytoplasmic component that translocates to the nucleus upon T-cell receptor (TCR) stimulation and an inducible nuclear component (31). Notably, the NFAT transcription complex is a primary molecular target of immunosuppressive drugs such as cyclosporine A. In immune cells, *NFATC1* typically exists in a highly phosphorylated state in the cytoplasm. Upon cellular stimulation, *NFATC1* undergoes dephosphorylation, facilitating its translocation into the nucleus (32). Research has indicated that PIAS1 may mitigate HIRI in mice via the *NFATC1* ubiquitination (33), suggesting that *NFATC1* may be involved in murine HIRI-related processes. Our study identified *NFATC1* as a key gene in LT-related HIRI datasets, found that it correlated with Wnt signaling scores and immune-related features, and confirmed its high expression in a murine liver IRI model. Future cell-specific intervention experiments are needed to clarify the functional role and mechanism of *NFATC1* in post-transplantation HIRI.

Thrombospondin-1 (*THBS1*), a member of the thrombospondin family, is an important regulator of platelet aggregation and angiogenesis. In normal tissues, it functions as a natural inhibitor of new blood vessel formation. *THBS1* can also modulate the adhesion, migration, and growth of endothelial cells, exerting either positive or negative effects. Interestingly, inhibition of the interaction between *THBS1* and its cell-surface receptor CD47 has been associated with an immune response in normal tissues during radiation therapy targeting tumors (34, 35). In this study, LT-related datasets showed that THBS1 was upregulated in post-reperfusion samples. GO and KEGG enrichment analyses indicated that THBS1 was associated with the biological process “response to interleukin-1.” Furthermore, TF-mRNA regulatory network analysis predicted that tumor protein 53 (TP53) might regulate THBS1 expression, suggesting that the TP53–THBS1 regulatory axis has potential research value in the pathophysiology of liver transplantation. Studies of *THBS1* expression and its regulatory mechanisms in colorectal liver metastasis further suggest that *THBS1* expression may change in response to hepatic pathological challenges, including tumor metastasis and adaptive processes after liver transplantation (36). Moreover, our study identified *THBS1* as a potential contributor to early post-transplant hepatic ischemia-reperfusion injury. Integrated transcriptomic analyses showed that THBS1 was associated with Wnt-related and immune-related signatures. Its significant upregulation at 6 hours in the non-transplant murine HIRI model further supported its expression change during the acute phase of ischemia-reperfusion injury, whereas pseudotime analysis suggested that THBS1 may be relatively active at an early cellular state. These findings indicate that THBS1 may represent a candidate molecule associated with HIRI progression, consistent with previous evidence showing its involvement in other liver diseases (35). Specifically, altered THBS1 expression has been observed under various hepatic pathological conditions, including liver transplantation-related contexts. Therefore, the present analysis provides preliminary evidence for the expression characteristics and potential biological relevance of THBS1 in HIRI after liver transplantation, although its precise functional role and therapeutic value require further experimental validation.

As the body’s primary immunological hub, the liver harbors a sophisticated immune repertoire comprising innate defenses, including Kupffer cells and natural killer cells, and adaptive regulators, including dendritic cells and natural killer T cells, that orchestrate hepatic immunosurveillance (37–39). These immune cells play critical roles in the progression of HIRI. Among them, the excessive inflammatory response driven by macrophages is considered a key contributor to HIRI. A distinctive feature of liver injury is the increased presence of macrophages within the liver, primarily resulting from blood monocyte infiltration and differentiation into monocyte-derived macrophages. Hepatic macrophages are generally categorized into M1 and M2 macrophages based on phenotypic and functional differences. M1 macrophages exacerbate inflammation, whereas M2 macrophages suppress it. Both macrophage types are instrumental in modulating sterile inflammation in the liver and play pivotal roles in the onset, progression, and resolution of HIRI (40).

We used TF-mRNA and lncRNA-miRNA-mRNA network analyses to investigate potential regulatory mechanisms involving the key genes. Through prediction analysis, we identified 10 related TFs, 70 miRNAs, and 39 lncRNAs. Previous research suggests that AP-1 and AP-1-dependent miRNAs collaboratively form a coregulatory network in which AP-1 target genes are tightly regulated. This finding enhances understanding of the mechanisms underlying HIRI and provides a potential approach for identifying therapeutic targets (41). In addition, modulation of miRNA levels may serve as a biomarker strategy for early diagnosis of HIRI (42). In mouse experiments, the lncRNA Hnf4αos was observed to mediate an inhibitory effect on PGC1α via the Hnf4αos/Hnf4α double strand, thereby exacerbating HIRI in mice (43). Moreover, TP53, a well-known tumor suppressor gene, was identified in this study as a predicted TF for *HSP90AB1* and *THBS1*, suggesting that TP53 may play a role in the progression of HIRI. In addition, *HSP90AA1* and *HSP90AB1* appeared to be involved in HIRI progression. The results of the present study suggest that early inhibition of *THBS1* could attenuate inflammatory responses, whereas delayed administration of HSP90 inhibitors might facilitate liver regeneration by modulating relevant signaling pathways (44). These findings provide preliminary insights for understanding HIRI-related mechanisms and may inform future therapeutic exploration in liver transplantation.

Single-cell sequencing analysis provided cellular-level interpretation of the four candidate key genes identified through bulk transcriptome screening. *HSP90AA1* and *HSP90AB1* were broadly expressed and relatively enriched in endothelial cells and macrophages. Given that endothelial injury is an important early event in HIRI-associated microcirculatory disturbance (45, 46) and that the HSP90 family may be involved in regulating hepatic sinusoidal endothelial cell dysfunction (47), we speculated that expression changes in HSP90-related genes might be associated with early endothelial injury. Meanwhile, HSP90 can assist in NLRP3 inflammasome assembly (48), which was consistent with our finding that M0 macrophages were positively correlated with HSP90 genes, suggesting their possible involvement in early proinflammatory responses. *THBS1* was predominantly expressed at high levels in macrophages and HSCs, and pseudotime analysis indicated that it was more active during early differentiation. Previous studies have shown that *THBS1* is associated with inflammatory sensing in macrophages and HSC activation (49, 50), suggesting its potential involvement in inflammatory responses and tissue remodeling. *NFATC1* was predominantly expressed at high levels in B cells, and B cells can participate in liver immune regulation via IL-10 (51); *NFATC1* is also associated with B-cell activation and differentiation (52). Therefore, single-cell analysis not only supplemented information on the cellular origins of the four genes but also provided clues for understanding their possible involvement in endothelial injury, inflammatory amplification, fibrosis-related responses, and immune regulation during HIRI. However, these inferences require validation in future functional experiments.

Functional enrichment analysis revealed that the four candidate key genes were primarily associated with pathways such as Jak-STAT signaling, chemokine signaling, and cytokine activity, consistent with the inflammatory background of ischemia-reperfusion injury after liver transplantation (53), suggesting that their expression changes may partially reflect the status of tissue inflammatory injury. Concurrently, expression levels of the four genes were positively correlated with the Wnt/β-catenin pathway score, indicating a potential link with Wnt pathway status. Previous studies have shown that *HSP90AB1* can interact with the Wnt co-receptor LRP5 and enhance canonical Wnt signaling (28); TSP-1, encoded by *THBS1*, can interact with HSPGs (54), which are involved in Wnt ligand distribution and signal-gradient regulation (55); moreover, GSK3β, the upstream kinase of *NFATC1*, is also a core component of the Wnt/β-catenin destruction complex (56, 57). Therefore, we speculated that these four genes may be situated at the intersection of inflammatory responses and Wnt/β-catenin signaling-related changes. However, this conclusion was based primarily on correlation analysis and indirect evidence from the literature; thus, a direct causal regulatory relationship between the Wnt pathway and the four genes cannot yet be established.

This study has several limitations. First, it was based primarily on public bulk transcriptomic, single-cell sequencing, and bioinformatics analyses to screen candidate genes, and experimental validation was relatively limited. Therefore, the current results mainly support expression changes and potential associations of *HSP90AA1*, *HSP90AB1*, *NFATC1*, and *THBS1* during HIRI but cannot establish their causal roles in LT-related HIRI. Second, the murine HIRI model mainly validated upregulation of the four genes at 6 hours after reperfusion, the peak of injury, whereas the public transcriptomic datasets may involve different post-transplantation or post-injury time points; this temporal heterogeneity may affect interpretation. Third, although the four genes were positively correlated with the Wnt/β-catenin pathway score, direct mechanistic experimental evidence is lacking; thus, this relationship should be interpreted as a potential association. Furthermore, the IRGs used in this study were mainly derived from the ImmPort database, and the WRGs were compiled based on classical pathway components, downstream effectors, and pathway cross-regulatory factors. Finally, the AUC results only suggest that these genes have discriminatory potential in the analyzed datasets and should not be equated with clinical diagnostic utility. Future studies should expand to multicenter clinical liver transplantation samples, incorporate dynamic sampling at defined reperfusion time points, use liver transplantation-specific animal models, include gene functional interventions and Wnt/β-catenin pathway modulation experiments, and perform sensitivity analyses using multiple gene set sources to further validate the temporal changes, functional roles, underlying mechanisms, and robustness of the screening results for the four candidate genes in LT-related HIRI.

## 5 Conclusion

By integrating bioinformatics analyses with expression validation in a non-transplant murine HIRI model, we identified *HSP90AA1*, *HSP90AB1*, *NFATC1*, and *THBS1* as key genes associated with LT-related HIRI and observed their upregulation during the acute phase of ischemia-reperfusion injury. Their stage-specific expression characteristics and correlations with Wnt/β-catenin pathway scores and immune responses provide clues for understanding HIRI-related transcriptional changes. The murine HIRI model results mainly support expression changes in these genes during ischemia-reperfusion injury but do not directly validate their functional roles in liver transplantation-associated HIRI. Future studies incorporating conditional knockout, pharmacological inhibition, or pathway intervention experiments in liver transplantation models are needed to further clarify their causal roles and therapeutic value.

## Data Availability

The datasets presented in this study can be found in online repositories. The names of the repository/repositories and accession number(s) can be found in the article/**Supplementary Material**.

## Supplementary material

Supplementary Figure 1 Full-length Western blot images.

Supplementary Figure 2 Single-cell cluster marker gene expression profiling.

Supplementary Figure 3 Marker gene expression validation of annotated single-cell types.

Supplementary Figure 4 Cell-cell ligand-receptor interaction network in membranous nephropathy.
